# Dual Regulation of Osteogenesis and Inflammation by Pomegranate ( *Punica granatum* L.) Extract in Periodontal Ligament-Derived Stem Cells: Implications for Regenerative Medicine

**DOI:** 10.1055/s-0045-1814461

**Published:** 2026-01-23

**Authors:** Safaa Baz, Heba Mahmoud, Shereen N. Raafat, Ali A.W. Kotb, Joudy Kamalah, Mai Hafez

**Affiliations:** 1 Department of Oral Pathology, Faculty of Dentistry, The British University in Egypt, Cairo, Egypt; 2 Department of Oral Biology, Faculty of Dentistry, The British University in Egypt, Cairo, Egypt; 3 Centre for Innovative Dental Sciences (CIDS), Faculty of Dentistry, The British University in Egypt (BUE), El Shorouk City, Egypt; 4 Department of Pharmacology, Faculty of Dentistry, The British University in Egypt (BUE), El Shorouk City, Egypt; 5 Department of Oral and Maxillofacial Pathology, Faculty of Dentistry, Cairo University, Giza, Egypt; 6 College of Dentistry, Gulf Medical University, Ajman, United Arab Emirates

**Keywords:** pomegranate, mesenchymal stem cells, osteogenic differentiation, anti-inflammatory activity, alizarin red staining, cell viability, regenerative medicine

## Abstract

**Objectives:**

This article aims to compare the osteogenic along with anti-inflammatory activity of different concentrations of pomegranate extract (PG) on human periodontal ligament-derived stem cells (hPDLSCs) in vitro.

**Materials and Methods:**

hPDLSCs were acquired from the root surface of removed molars, cultured, and then distinguished by flow cytometry analysis and several lineage differentiation potentials. To identify the effect of PG on hPDLSCs' viability, an MTT assay was performed. hPDLSCs were maintained in osteogenic induction medium with varying concentrations of PG. At the end of the induction period, osteogenesis was assessed using Alizarin Red staining, ALP assay, and qRT-PCR to determine the expression of OPG, RUNX2, Ki67, and TNF-α.

**Statistical Analysis:**

All experiments were conducted in triplicate, and data are presented as the mean ± standard deviation (SD). One-way ANOVA followed by Tukey's post hoc test was performed to assess statistical significance at a threshold of *p* < 0.05.

**Results:**

The results of the MTT assay demonstrated that 100 mg/mL PG had significantly lower cell viability than the other concentrations. Statistical analysis of the ALP enzyme activity was mostly pronounced at 6.25 and 12.5 mg/mL concentrations, while it was least pronounced at 50 mg/mL. PCR revealed that the group treated with a 6.25-mg/mL concentration exhibited significantly elevated expression rates of RUNX2, OPG, and Ki67 in contrast with the control group. Conversely, the 50-mg/mL concentration group demonstrated the lowest expression levels. Regarding TNF-α, the 50-mg/mL concentration group showed the greatest expression levels compared with the control group and all other concentrations.

**Conclusion:**

The data indicate that low concentrations of PG could enhance osteogenic differentiation and exert anti-inflammatory effects on hPDLSCs. These dual actions suggest that PG, at optimized doses, may serve as a promising natural agent for periodontal regeneration and bone tissue engineering. Future studies are warranted to evaluate its clinical potential in regenerative medicine.

## Introduction

In the regenerative medicine field, a primary objective is the identification and optimization of drugs that can potentiate the regenerative processes and differentiation of mesenchymal stem cells (MSCs) toward the desired cell type. 1 While MSCs have a strong capacity to develop into osteoblasts, stem cell therapy has become increasingly popular in clinical settings over the years for the treatment of bone disorders. 2

Stem cell differentiation and osteogenesis can be promoted by treatment with bone-associated factors. Lately, research on both natural and artificial compounds has received increasing attention. Numerous herbs possess compounds that can promote the osteogenic differentiation of MSCs, thereby facilitating bone production. 3 It has been demonstrated that polyphenolic chemicals, including flavonoids, have an impact on promoting osteoblasts formation both in vitro and in vivo. 4 5

Pomegranate ( *Punica granatum* L.) (PG) is a rich source of micronutrients and is considered an attractive medicinal agent due to its wide range of therapeutic applications. 6 7 Furthermore, numerous extracts from different parts of this plant exhibit several biological actions, including antitumor, 7 antibacterial, 8 and antifungal properties. 9 Moreover, PG is increasingly recognized for its significant antioxidant properties, 10 owing to its rich content of hydrolysable tannins (such as gallic and ellagic acid), flavonoids (including anthocyanins, catechins, as well as various complex flavonoids), as well as polyphenols like punicalagin. 11

Human periodontal ligament-derived stem cells (hPDLSCs) are a promising resource for regenerative therapies and tissue engineering. Additionally, they have lower immunoreactivity and can be easily acquired using noninvasive procedures, in contrast to bone marrow MSCs. 12

The objective of this study was to evaluate how different concentrations of PG extract affect the osteogenic and anti-inflammatory properties of hPDLSCs in vitro.

## Materials and Methods

This in vitro study was conducted at the Stem Cell Center, Research Centre of Excellence, Dental Group, Faculty of Dentistry, The British University in Egypt. Ethical approval was obtained from the Faculty of Dentistry's Ethical Committee at the British University in Egypt (registration number: FD BUE REC 24–068).

### Isolation and Culture of hPDLSCs

Stem cells isolation and culture were performed according to a previously reported method. 13 Permanent human molars ( *n* = 3) were procured from healthy donors at the Department of Maxillofacial Surgery for orthodontic purposes. The patients consented to follow a comprehensive explanation of the study objectives provided by the principal investigator. The extracted molars were intact, devoid of any carious lesions, and there were no signs of gingival inflammation.

Periodontal ligament tissues isolated from the root surfaces of extracted molars were cultured using the outgrowth method. For this procedure, Dulbecco's modified eagle's medium/nutrient medium/F12 Ham (DMEM/F12; Catalog # 2715699, Gibco BRL, California, United States) was enriched via 10% fetal bovine serum (FBS; Catalog # 2575628, Gibco BRL) and 1% antibiotics. The antibiotic mixture comprising 300 U/mL penicillin and 300 mg/mL streptomycin was obtained from Capricorn Scientific (Catalog # PS-B, Capricorn Scientific GmbH, Ebsdorfergrund, Germany). The explants were incubated in a humidified atmosphere containing 5% CO _2_ at 37 °C. Using an inverted microscope, cell growth and morphology were assessed. Cells were collected for future storage at passage 3 (P3), and in subsequent experiments, cells at passage 4 (P4) were utilized.

### Characterization of the hPDLSCs

Characterization of the hPDLSCs was performed using flow cytometry and multiple lineage differentiation to identify the isolated cells. 14

#### Flow Cytometry

Trypsin/Ethylenediaminetetraacetic acid (EDTA; Catalog # E5134, Sigma-Aldrich, St. Louis, Missouri, United States) solution was used to separate the cultured cells at P4 and produce a suspension of individual cells. Subsequently, the cells were centrifuged, and cell pellets were resuspended and stained with primary antibodies conjugated to a fluorescent dye in sterile phosphate-buffered saline (PBS; Catalog # P4417, Sigma-Aldrich) containing 2% FBS in the dark for 45 minutes. The used fluorescein-labeled monoclonal antibodies were specific to CD34, CD45, human leukocyte antigen-DR (HLA-DR), CD73, CD90, and CD105 (BD Biosciences, Piscataway, New Jersey, United States). A flow cytometer (FACSCalibur; BD Biosciences) was employed to conduct the analysis.

#### Multilineage Differentiation

To determine the adipogenic, chondrogenic, and osteogenic differentiation potential of the isolated hPDLSCs, multilineage differentiation was conducted utilizing a validated commercial human mesenchymal stem cell functional identification kit (Catalog # SC006, R&D Systems Inc., Minnesota, United States). Oil Red O (Catalog # O0625, Sigma-Aldrich) and Alcian Blue (A5268, Sigma-Aldrich) were sourced.

### Pomegranate Extract (PG)

Pomegranate (PG; *Punica granatum* L.) extract was obtained from BulkSupplements.com (ASIN B00O3AFURQ, Henderson, Nevada, United States).

### MTT Assay

The 3-(4,5-dimethylthiazol-2-yl)-2,5-diphenyltetrazolium bromide (MTT) assay was utilized to evaluate the effect of PG extract on the viability of hPDLSCs. 10 × 10 ^3^ cells per well were cultured into 96-well plates with serial dilutions of the PG (100, 50, 25, 12.5, and 6.25 mg/mL). On days 1, 3, and 7, a total of 100 µL of MTT reagent (0.5 mg/mL; Catalog # M5655, Sigma-Aldrich) was applied to the cells in each group, and cells were incubated for 4 hours. The formed violet color of the insoluble formazan crystals was solubilized by the addition of 100 µL of dimethyl sulfoxide (DMSO; Catalog # D2650, Sigma-Aldrich), and then the produced color was determined using a spectrophotometer at a wavelength of 450 nm. 15 16 The percentage of living cells was determined using the following equation:



### In Vitro Differentiation Assays

Different concentrations of PG were added to the osteogenic induction medium, including 50, 25, 12.5, and 6.25 mg/mL. The negative control group comprised cells cultured in conventional DMEM/F12 supplemented with 10% FBS. The positive control comprised cells grown exclusively in osteogenic differentiation media, including standard culture media augmented with 10 mmol/L β-glycerophosphate (Catalog # G9422, Sigma-Aldrich), 10 nmol/L dexamethasone (D4902, Sigma-Aldrich), and 50 μg/mL ascorbic acid (Catalog # A8960, Sigma-Aldrich). 17

#### Alizarin Red Staining and Quantification

Following 2 weeks of osteogenic induction and the appearance of mineralized nodules, the Alizarin Red assay was performed. Briefly, cells were fixed for 20 minutes in 70% ethanol. After 20 minutes of staining with Alizarin Red S stain (Catalog # A5533, Sigma-Aldrich), the cells were washed five times using water, following the manufacturer's directions. The stained mineralized nodules were observed utilizing an inverted microscope. Thereafter, 10% warm acetic 18 19 was used to solubilize the stain, and the resulting color was quantified with a spectrophotometer at 490 nm.

#### Alkaline Phosphatase Activity

Monolayers were washed twice with PBS to assess alkaline phosphatase (ALP) activity of the enzyme, followed by an additional wash using 0.5 mL ALP buffer (ALPB; Catalog # A9226, Sigma-Aldrich). Each well received 250 μL of ALPB and an identical volume of p-nitrophenyl phosphate disodium salt solution (p-NPP; Catalog # N2765, Sigma-Aldrich) pre-cooled to 4 °C. The chemical reaction in each well was immediately stopped by removing 50 μL and mixing it with the same volume (50 μL) of NaOH (0.3 M). The preceding procedure was performed once every minute for 10 minutes. The ALP enzyme produced by the cells converted colorless p-NPP into yellow para-nitrophenolate. A spectrophotometer was used to measure the absorbance at 405 nm. The rate of accumulation of yellow para-nitrophenolate (p-NP) was graphed, and the slope for each sample's response was calculated as detailed in a prior work. 20

### Quantitative Real-Time Reverse Transcription-Polymerase Chain Reaction

Bone-related markers, including osteoprotegerin (OPG), Runt-related transcription factor 2 (RUNX2), Ki67, and tumor necrosis factor-α (TNF-α), were analyzed via quantitative real-time reverse transcription-polymerase chain reaction (qRT-PCR) to assess differentiation potential. hPDLSCs (10 ^5 cells/well^ ) were plated in six well plates. Following a 2-week incubation period, reverse transcriptase was used to extract total RNA and transcribe it into complementary DNA. Following the manufacturer's directions, the RNeasy Mini Kit (Catalog # 160048808, Qiagen, Hilden, Germany) was used for RNA extraction. Reverse transcription was conducted using SYBR Green PCR Master Mix (Catalog # S4438, Sigma-Aldrich) on a Corbett Rotor-Gene 5-PLEX rotary analyzer (Software v1.7, Build 87, Qiagen).

To assess relative gene expression, the 2 − ΔΔCT technique was employed. Gene expression levels were normalized to the housekeeping gene, glyceraldehyde-3-phosphate dehydrogenase (GAPDH). The average and standard deviation of three separate triplicate trials were used to represent the data. Table 1 displays the gene primer sequence. 21

**Table 1:** Sequence of primers of determined genes

Marker	Forward	Reverse
OPG	CTAATTCAGAAAGGAAATGC	GCTGAGTGTTCTGGTGGACA
RUNX2	GTTATGAAAAACCAAGTAGCCAGGT	GTAATCTGACTCTGTCCTTGTGGAT
Ki67	AGAAGAAGTGGTGCTTCGGAA	AGTTTGCGTGGCCTGTACTAA
TNF-α	ATGTTGTAGCAAACCCTCAAGC	AGGACCTGGGAGTAGATGAGG
GAPDH	GGAGCGAGATCCCTCCAAAAT	GGCTGTTGTCATACTTCTCATGG

Abbreviations: GADPDH, glyceraldehyde-3-phosphate dehydrogenase; OPG, osteoprotegerin; RUNX2, runt-related transcription factor 2; TNF-α, tumor necrosis factor-α.

### Statistical Analysis

The conclusions of the triplicate experiments are presented as mean ± standard deviation (SD). Data normality was measured utilizing the Shapiro–Wilk test. Tukey's post hoc test was employed to perform a one-way analysis of variance (ANOVA) at a significance threshold of *p* < 0.05 to evaluate the statistical significance across the experimental groups. Statistical analysis was conducted using GraphPad Prism 9.0 (GraphPad Software).

## Results

### Stem Cell Isolation and Culture

hPDLSCs were effectively separated and proficiently grown. Under an inverted light microscope, the cells exhibited flexible adhesion with the characteristic spindle morphology of stem cells ( Fig. 1 ).

**Fig. 1:**
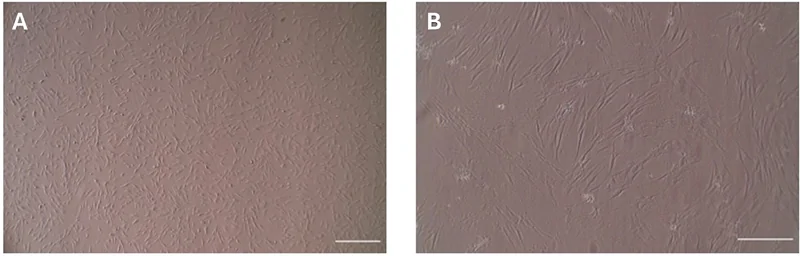
Representative images of isolated hPDLSCs. ( **A** ) Cells at passage 3, original magnification 40 × , scale bar 500 nm. ( **B** ) Cells at passage 4. Original magnification: 100 × , scale bar: 250 nm.

### Characterization

#### Flow Cytometry

The cells used for immunophenotypic investigation were those from passage four. The findings indicated that hPDLSCs were positive for the MSC markers CD73 (99.8%), CD90 (99.7%), and CD105 (99.15%), whereas they were almost negative (< 2%) for the hematopoietic and endothelial stem cell markers CD45 (0.31%), CD34 (0%), and HLA-DR (0.58%) ( Fig. 2A–F ).

**Fig. 2:**
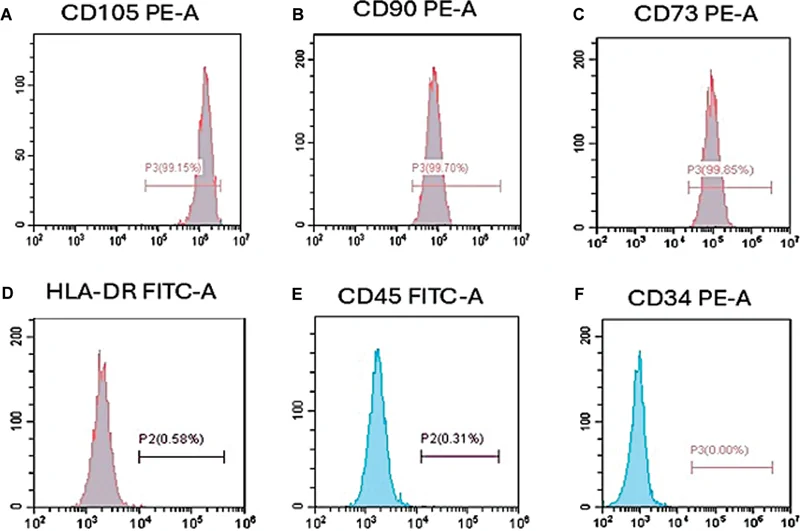
Immunophenotyping of isolated cells by flow cytometry. ( **A–C** ) The cells exhibited positive expression of mesenchymal stem cell markers CD105, CD90, and CD73 (> 95%); ( **D-F** ) negative expression (< 2%) of hematopoietic and endothelial markers HLA-DR, CD45, and CD34.

#### Multilineage Differentiation

Trilineage differentiation of hPDLSCs into osteogenic, adipogenic, and chondrogenic lineages was confirmed by histochemical stains. Alizarin red staining confirmed the presence of mineralized nodules, oil red staining verified the presence of oil droplets, and Alcian blue staining demonstrated the presence of proteoglycans, indicative of chondrogenic differentiation ( Fig. 3 ).

**Fig. 3:**
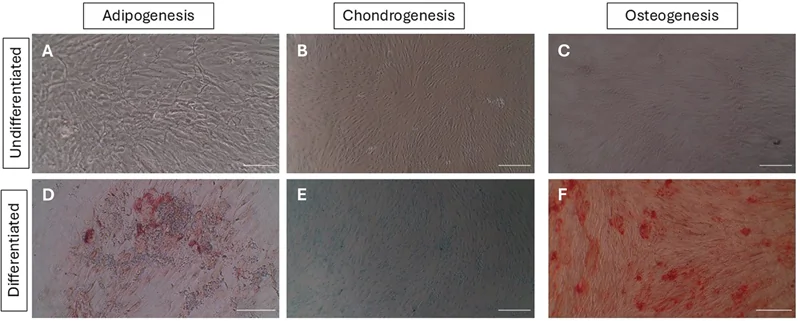
Microscopic images of hPDLSCs with undifferentiated controls ( **A–C** ) and differentiated cells ( **D–F** ) for adipogenesis ( **A, D** ), chondrogenesis ( **B, E** ), and osteogenesis ( **C, F** ). Original magnification: 100 × , scale bar: 250 nm.

### Cell Viability

The impact of different concentrations of PG on the viability of hPDLSCs was evaluated by the MTT assay. The findings demonstrated that each concentration of PG resulted in distinct levels of cell viability ( Fig. 4 ). Cells exposed to 100 mg/mL exhibited the lowest viability on days 1, 3, and 7, with viability percentages of 76.3, 67.19, and 65.82%, respectively. In contrast, cells treated with other diluted concentrations showed higher viability percentages (>70%) at the specified time points. Statistical analysis revealed that the 100-mg/mL concentration had significantly lower cell viability than the other concentrations, particularly on day 7 (viability below 70%, *p* < 0.05). Consequently, for subsequent studies, only the following concentrations were chosen: 50, 25, 12.5, and 6.25 mg/mL.

**Fig. 4:**
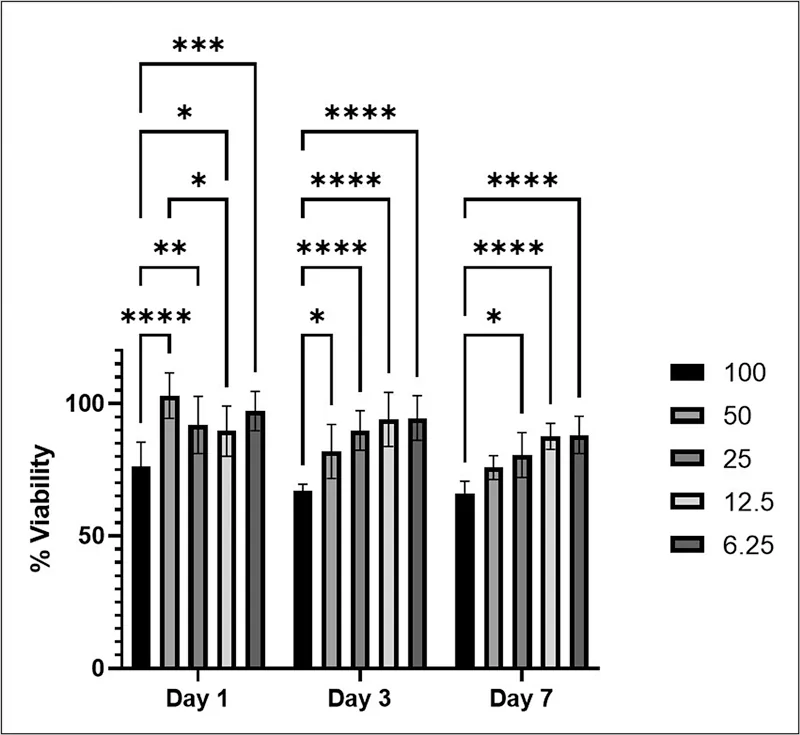
MTT assay results. hPDLSCs were cultured with varying concentrations of PG, and % viability was determined on days 1, 3, and 7. Data are presented as the mean % viability ± SD. The samples were performed in triplicate ( *n* = 3).

### Alizarin Red Assay

To identify calcified nodules, Alizarin red was used to stain the cultured cells after osteogenic differentiation. The stained mineralized nodules were more prevalent at all PG concentrations in comparison to the positive control group (Osteo-group), exhibiting the greatest abundance at 6.25 mg/mL concentration and the lowest at 50 mg/mL ( Fig. 5A–F ). Statistical analysis confirmed that the stain density was greater at the diluted concentrations, with the highest absorbance recorded at 6.25 mg/mL ( *p* < 0.05) in comparison to other concentrations. The mean absorbance values of the solubilized stain at 50, 25, 12.5, and 6.25 mg/mL were 0.47 ± 0.01, 0.57 ± 0.05, 0.56 ± 0.02, and 0.64 ± 0.01, respectively ( Fig. 5G ).

**Fig. 5:**
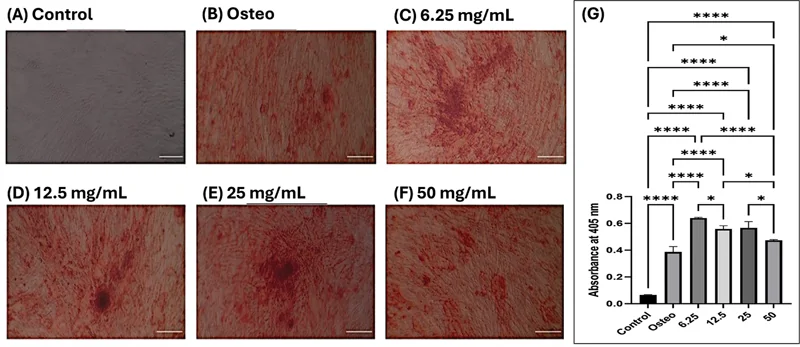
Alizarin red assay results. ( **A–F** ) Images of mineralized nodules after osteogenic induction. Original magnification: 100 × , scale bar: 250 nm. ( **G** ) Statistical analysis of the absorbance of the solubilized stain. The outcomes are shown as mean ± SD. The experiment was conducted three times independently ( *n* = 3).

### ALP Assay Results

The ALP kinetic profile exhibited a time-dependent elevation in the yellow p-NP product across various experimental treatments. The control group demonstrated the least accumulation rate, whereas concentrations of 12.5 and 6.25 mg/mL displayed the highest rates ( Fig. 6A ). The slope of each curve was calculated to determine the reaction rate, reflecting the ALP enzyme activity within the study groups. Statistical analysis of these slopes revealed that ALP enzyme activity was most pronounced at 6.25 and 12.5 mg/mL concentrations, while it was least pronounced at 50 mg/mL ( *p* < 0.01; Fig. 6B ).

**Fig. 6:**
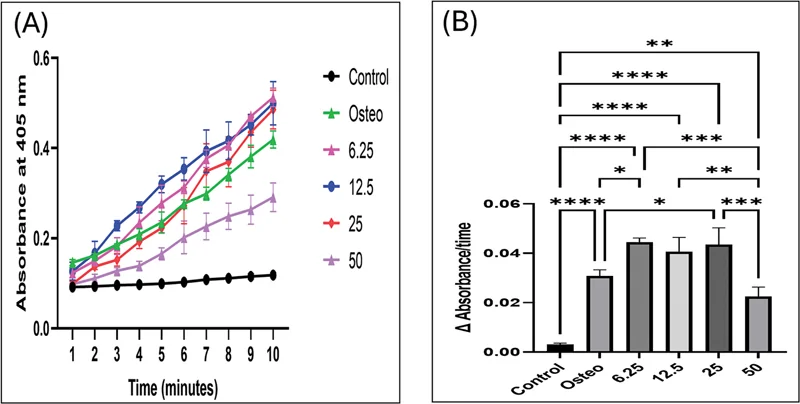
ALP assay results. ( **A** ) The kinetics of ALP enzyme activity were assessed by plotting the absorbance of the yellow p-NP product over time. ( **B** ) The slope of each reaction was calculated to determine the rate of the reaction, representing the ALP activity in every group. The outcomes are shown as mean ± SD. The samples were analyzed in triplicate ( *n* = 3).

### PCR Results

The expressions of osteogenic markers RUNX2 and OPG, the proliferation marker Ki67, and the proinflammatory marker TNF-α were evaluated with RT-qPCR. Results revealed that treatment with 6.25 mg/mL exhibited significantly elevated expression of RUNX2, OPG, and Ki67 compared with the control group and the groups treated with 12.5, 25.0, and 50.0 mg/mL amounts ( *p* < 0.01). Conversely, the group treated with 50 mg/mL demonstrated the lowest expression levels ( Fig. 7A–C ). Regarding TNF-α, the 50 mg/mL group showed the highest expression levels in comparison with the control and all other concentrations ( *p* < 0.001) ( Fig. 7D ).

**Fig. 7:**
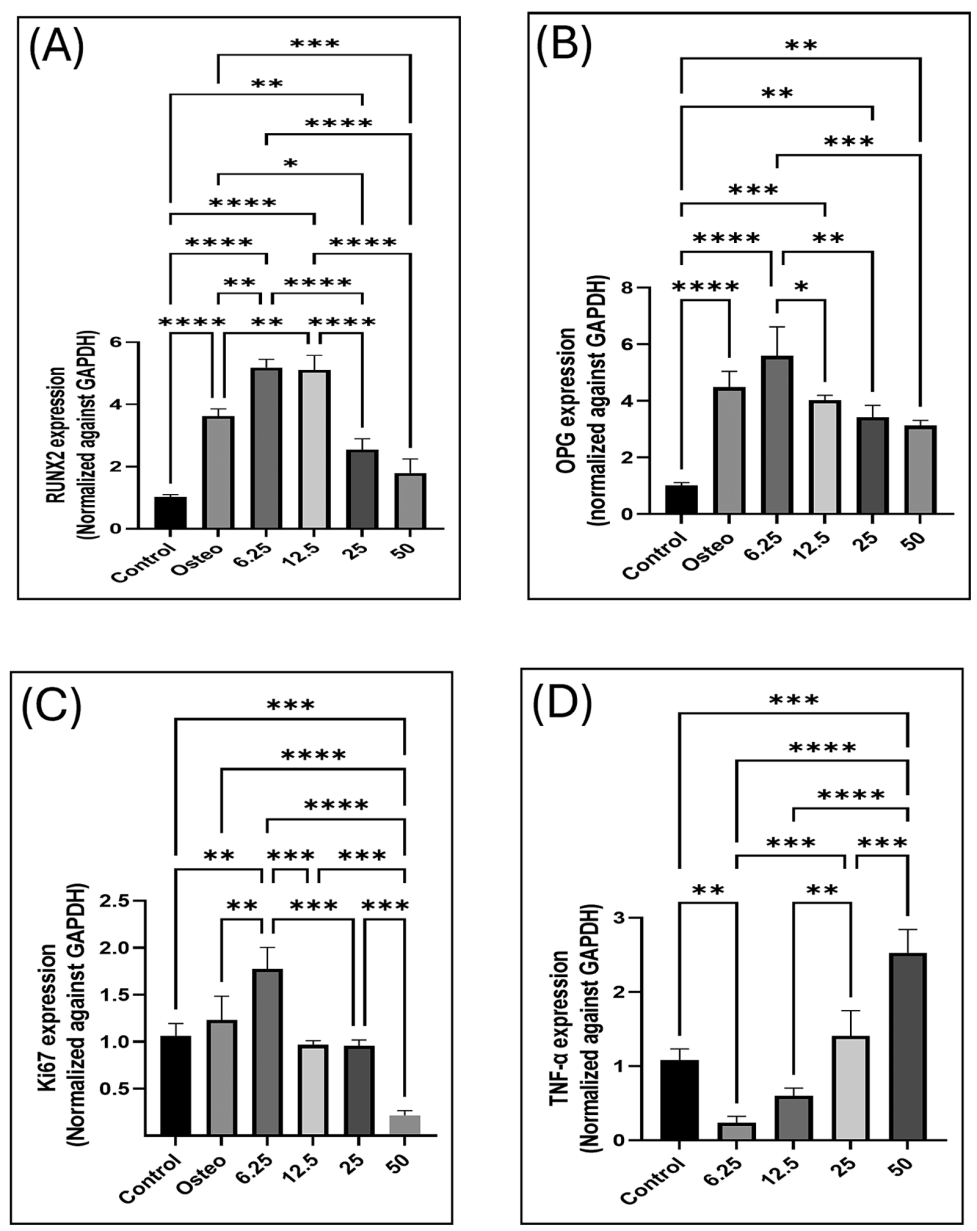
Osteogenic, proliferation, and proinflammatory markers expression using RT-qPCR. ( **A** ) Expression of RUNX2 osteogenic marker, ( **B** ) expression of OPG osteogenic marker, ( **C** ) expression of Ki67 proliferation marker, ( **D** ) expression of TNF-α proinflammatory marker. Data are presented as mean ± SD. Samples were analyzed in triplicate ( *n* = 3).

## Discussion

Osteogenic differentiation can be enhanced by bone-associated factors, with growing interest in natural and synthetic compounds. Herbal medicines, rich in phytochemicals, have shown the potential to promote stem cell differentiation and bone formation. 3

This study aimed to assess the effects of varying doses of the PG extract on hPDLSCs, focusing on the anti-inflammatory properties and osteogenic differentiation potential.

The current study revealed that hPDLSCs exposed to higher PG concentrations showed reduced cell viability, with the 100-mg/mL group displaying the lowest levels. In contrast, lower concentrations (6.25–25 mg/mL) maintained cell viability above 70% across days 1, 3, and 7, indicating higher cell viability at these doses.

Accordingly, it was found that high concentrations of PG extract led to reduced cell viability in mouse fetal mesenchymal cells and certain cell lines. They attributed the cause to oxidative DNA damage and high antiproliferative activity. 22 Sequentially, it could be used for the treatment of cancer, as reported in several studies suggesting that the whole PG fruit, along with its juice and oil, exhibits bioactive properties, including anti-inflammatory, antiproliferative, and antitumorigenic properties. 23 24 25

Alizarin red staining showed that calcified nodule formation was highest at 6.25 mg/mL PG extract and lowest at 50 mg/mL. ALP assays revealed minimal activity in the control group, while 6.25 and 12.5 mg/mL treatments induced the highest ALP activity. Increased Alizarin red staining intensity or ALP activity is indicative of elevated calcium deposition, which is often associated with bone growth or calcification. 26 27 This may be attributed to the high estrogenic chemicals and phenolic content, including ellagitannins, anthocyanins, and ellagic acid, in conjunction with essential minerals (e.g., potassium, magnesium, and copper of PG extract) that may facilitate differentiation and proliferation of osteoblasts while inhibiting osteoclast activity. 28 29 Also, PG contains phytoestrogens, including genistein and daidzein, which increase osteoblast activity, including ALP production. 30

Moreover, Spilmont et al found that all components of pomegranate (PG) effectively prevented ovariectomy-induced bone loss in mice, likely due to their ability to reduce oxidative stress and suppress inflammation. 31

In contrast, research was performed to evaluate the impact of high-dose PG extract on oxidative stress and inflammation in rats following sepsis induction. It was concluded that the pretreatment with high doses of PG before sepsis induction impaired the critical early neutrophil response following sepsis onset, thereby exacerbating oxidative stress. 32 This may elucidate the findings of the current investigation, indicating that elevated doses of PG diminished bone growth due to an increase in oxidative stress.

Osteogenic markers, including RUNX2 and OPG, were analyzed by qRT-PCR to evaluate their differentiation potential. Runx2 is considered the primary transcription factor responsible for the initial phases of osteogenesis and the subsequent process of bone mineralization. Furthermore, during the early stages of osteoblast differentiation, it activates essential genes involved in bone matrix production. 33 OPG is a glycoprotein that controls bone remodeling by binding to (RANKL), preventing its interaction with (RANK). This inhibits osteoclast differentiation and activity, resulting in increased bone mass and strength. 34

In the ongoing study, the qRT-PCR results of osteogenic RUNX2 and OPG expression revealed that the groups treated with 6.25, 12.5, 25, and 50 mg/mL concentrations showed significantly elevated expression levels of both compared with the control group. Conversely, the 50-mg/mL group showed the lowest expression levels. These results are consistent with previous studies that found that PG extract (10–100 μg/mL) promoted morphological changes, matrix mineralization, and increased RUNX2 expression in rat osteoblasts in a dose-dependent manner. These effects may be attributed to β-sitosterol and flavonoids in PG, which enhance RUNX2 expression and activity. 35

Concerning the Ki67, a nuclear proliferative marker and cell cycle regulatory protein, it was reported that the group treated with 6.25, 12.5, 25.0, and 50.0 mg/mL concentrations showed significantly elevated expression levels of Ki67 compared with the control. Conversely, the group treated with 50 mg/mL demonstrated the lowest expression. This could be attributed to the presence of a high estrogenic environment, which in turn shortened the cell cycle, leading to cells moving from the G0 to the G1 phase. This is succeeded by an increase in the number of cells undergoing division and progressing through G1 and S phases. 36 This is consistent with the findings of Celiksoy et al, who reported that high concentrations of PG rind extract significantly reduced fibroblast proliferation due to cytotoxic effects, whereas lower concentrations enhanced fibroblast regeneration and promoted wound healing. 37

Regarding TNF-α, a key modulator of inflammation, the 50-mg/mL concentration group revealed higher expression levels compared with the control group and all other concentrations. The current findings are aligned with those of Liu et al, who stated that punicalagin, the primary ingredient of PG polyphenol, significantly reduced TNF-triggered inflammatory activation in human umbilical vein endothelial cells. It inhibited monocyte adherence to the endothelial layer by inhibiting nuclear factor kappa light-chain enhancer of activated B cell (NF-κB) signaling by suppressing IκB kinase (IKK) activation and reducing the expression of adhesion markers. 38 Additionally, PG reduced the levels of inflammatory mediators, including TNF-α, interleukin-6 (IL-6), cyclooxygenase-2 (COX-2), and NF-κB. This anti-inflammatory effect contributes to its protective properties against both natural and chemical mediators. 39

The study showed that low PG extract concentrations enhanced osteogenesis and reduced inflammation, while higher concentrations were cytotoxic. Similarly, Read et al reported that high intake of PG fruit led to cattle deaths due to its toxic metabolite contents, such as gallic acid, ellagic acid, and punicalagin, which caused cytotoxic effects on bovine kidney epithelial cells. 40

## Conclusion

The findings of this study indicated that low concentrations of PG enhanced osteogenic differentiation and exerted anti-inflammatory effects on (hPDLSCs) in vitro. In contrast, higher concentrations decreased viability and promoted inflammation, indicating that PG may serve as a promising natural agent for periodontal regeneration and bone tissue engineering. However, the optimal dosage must be carefully determined, and further research is needed to determine safe and effective dosages for clinical use in regenerative medicine.
